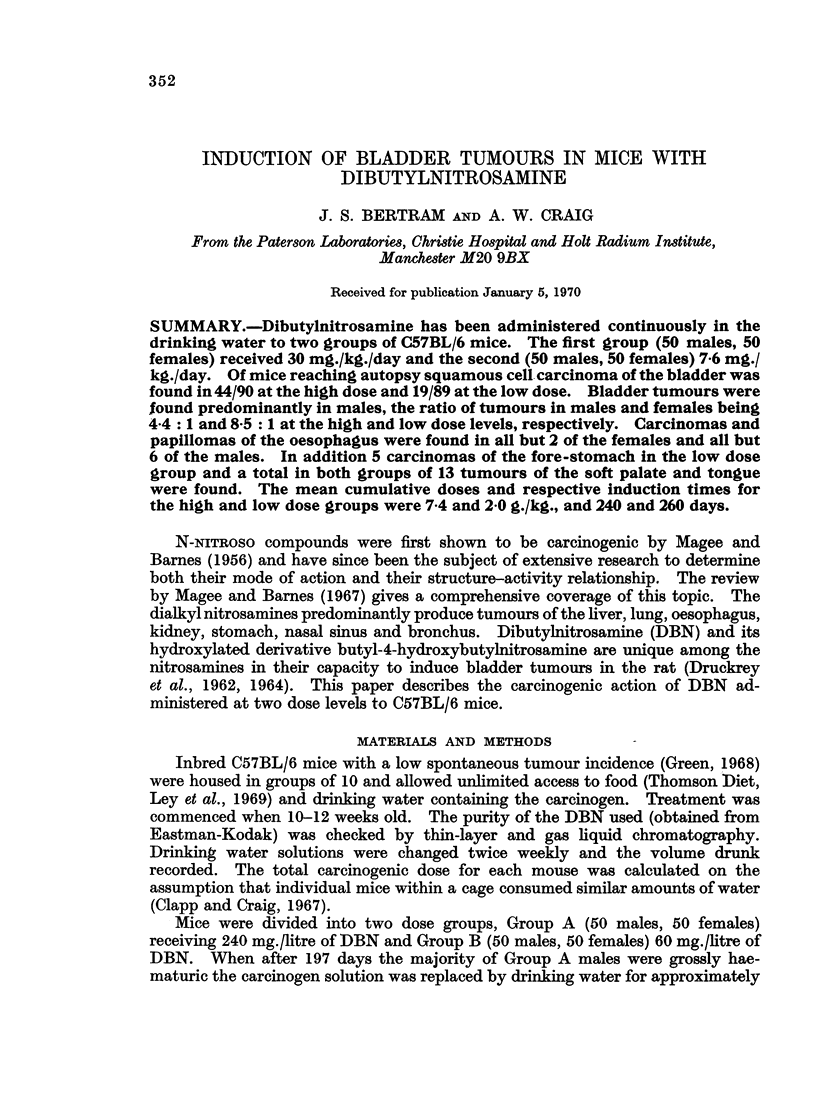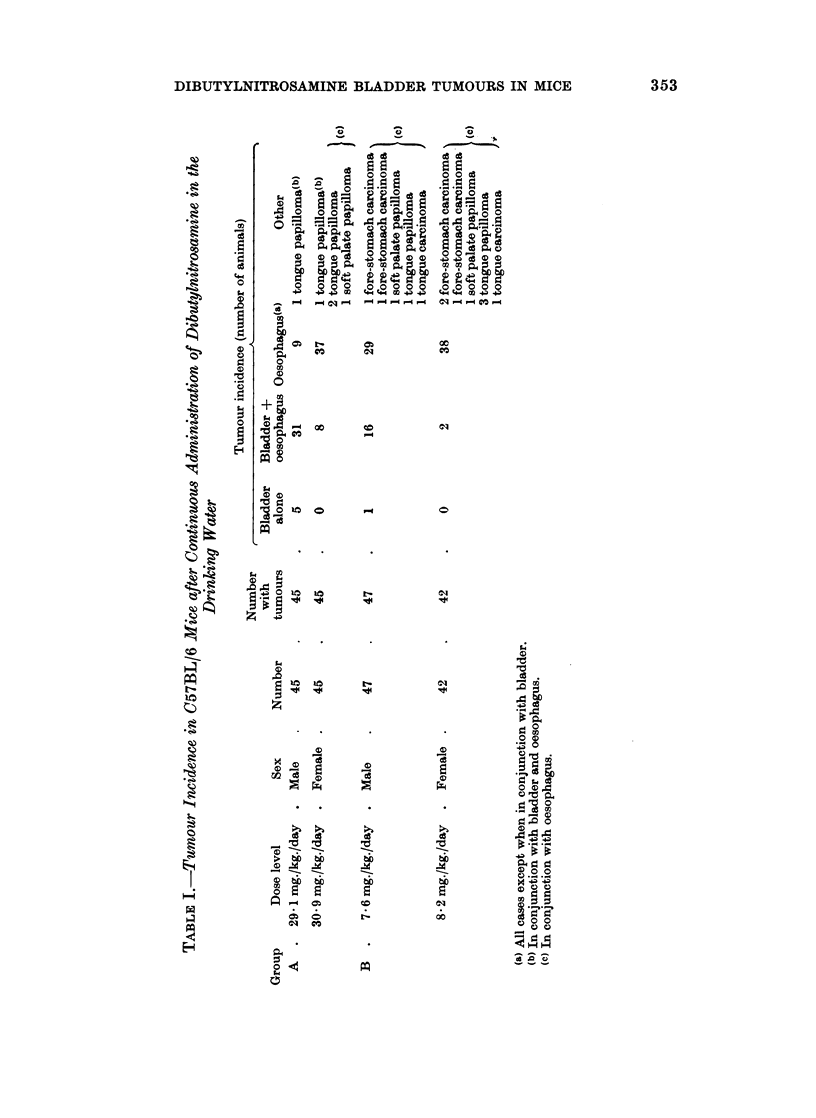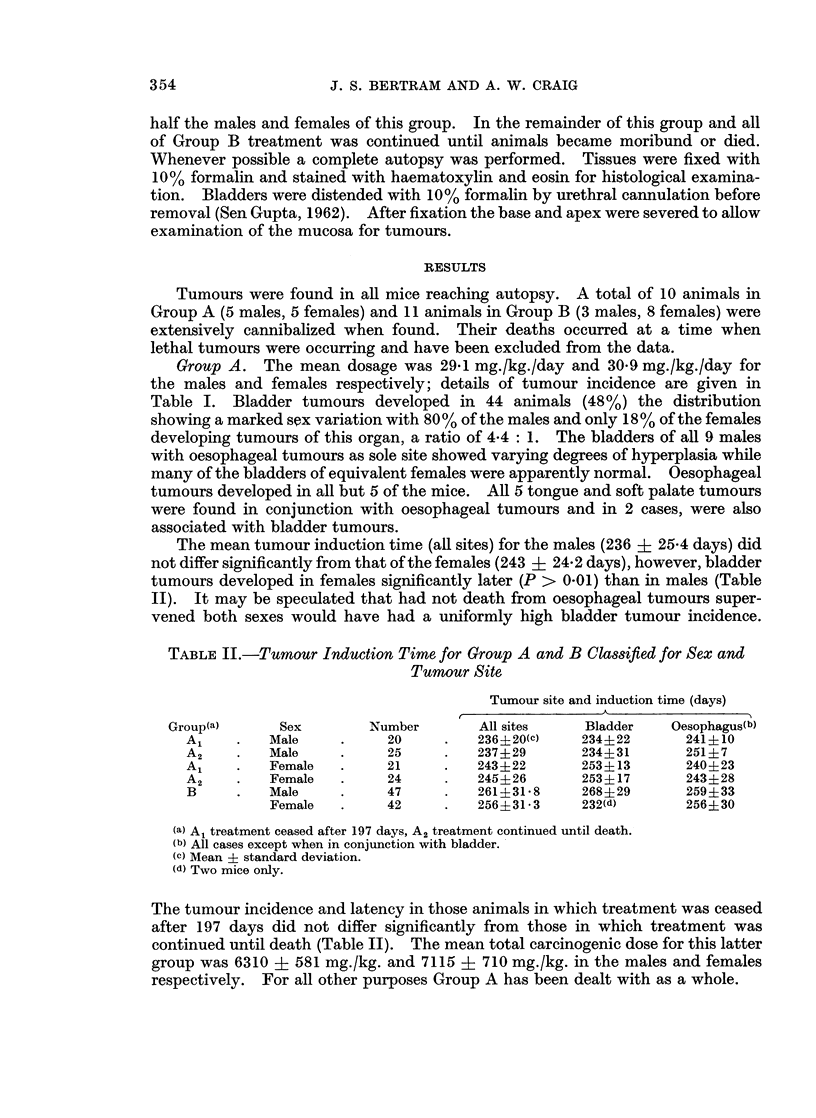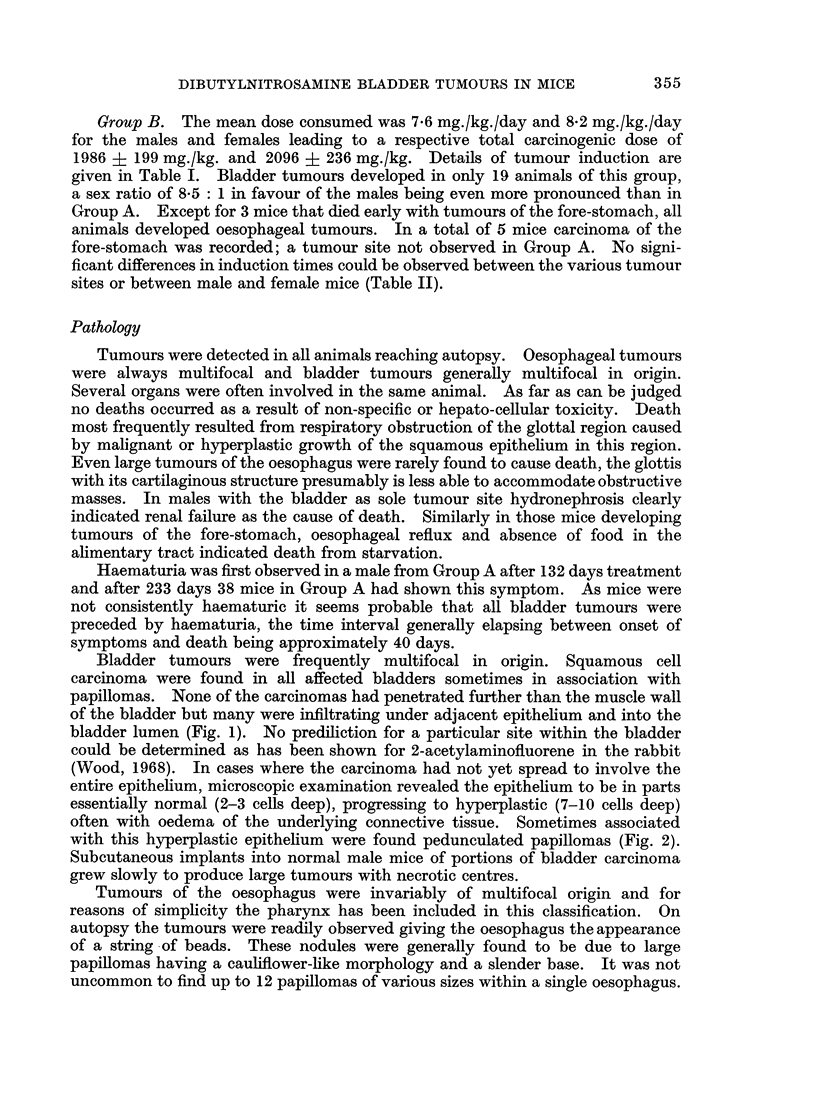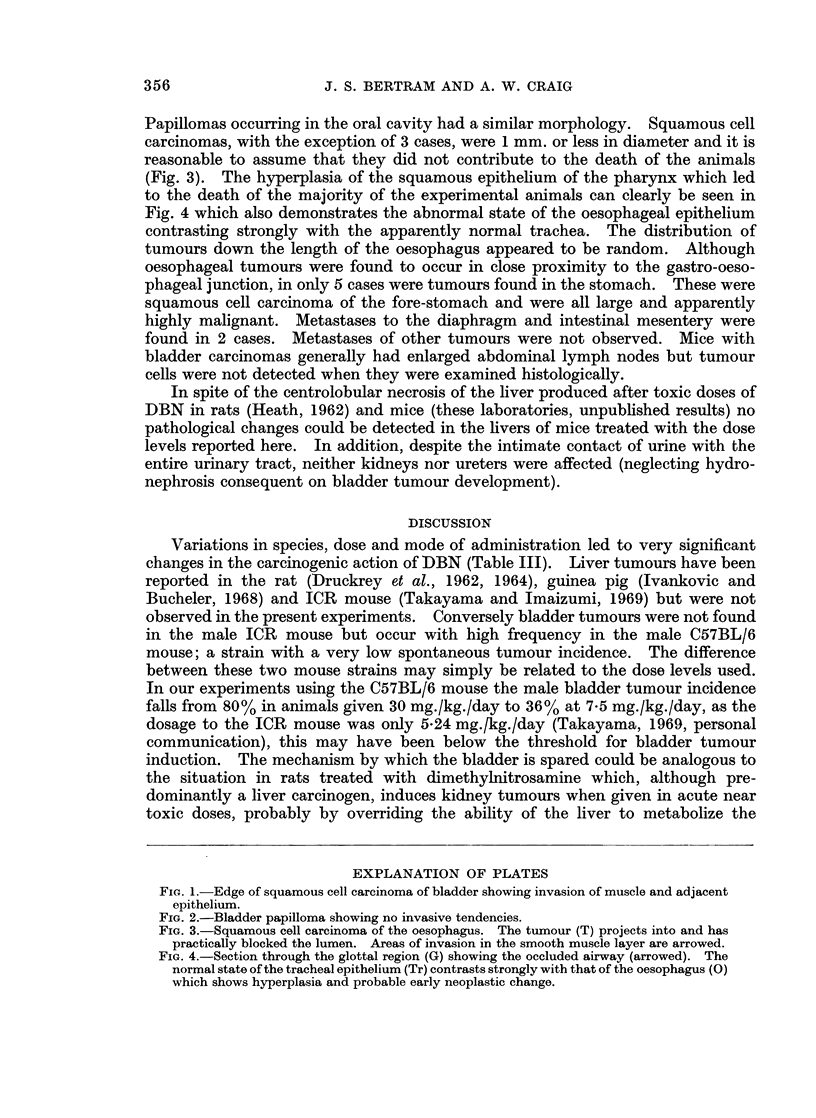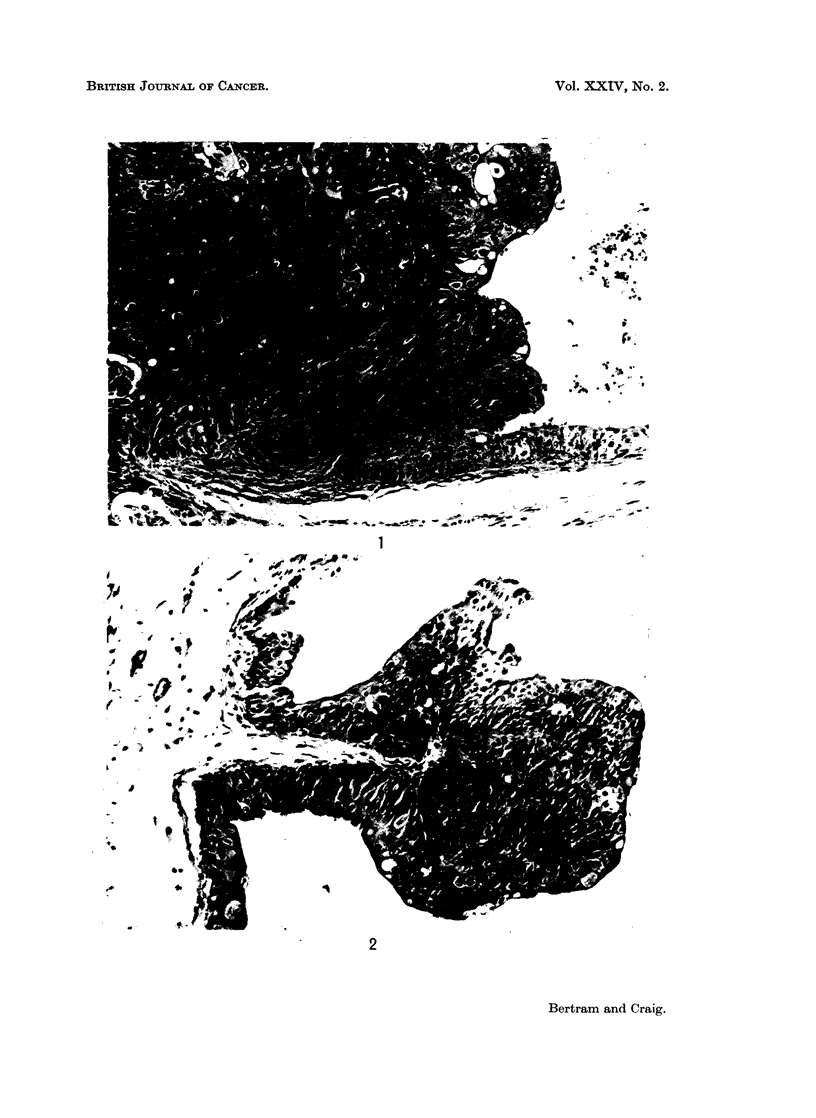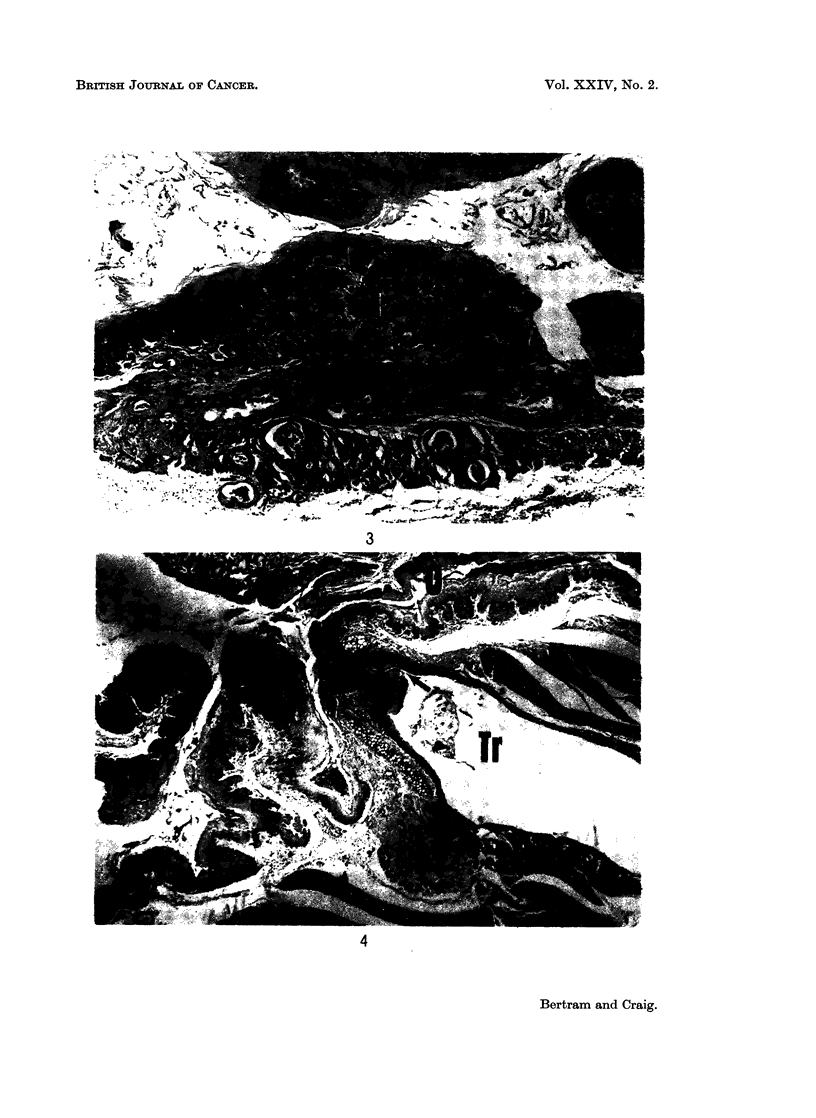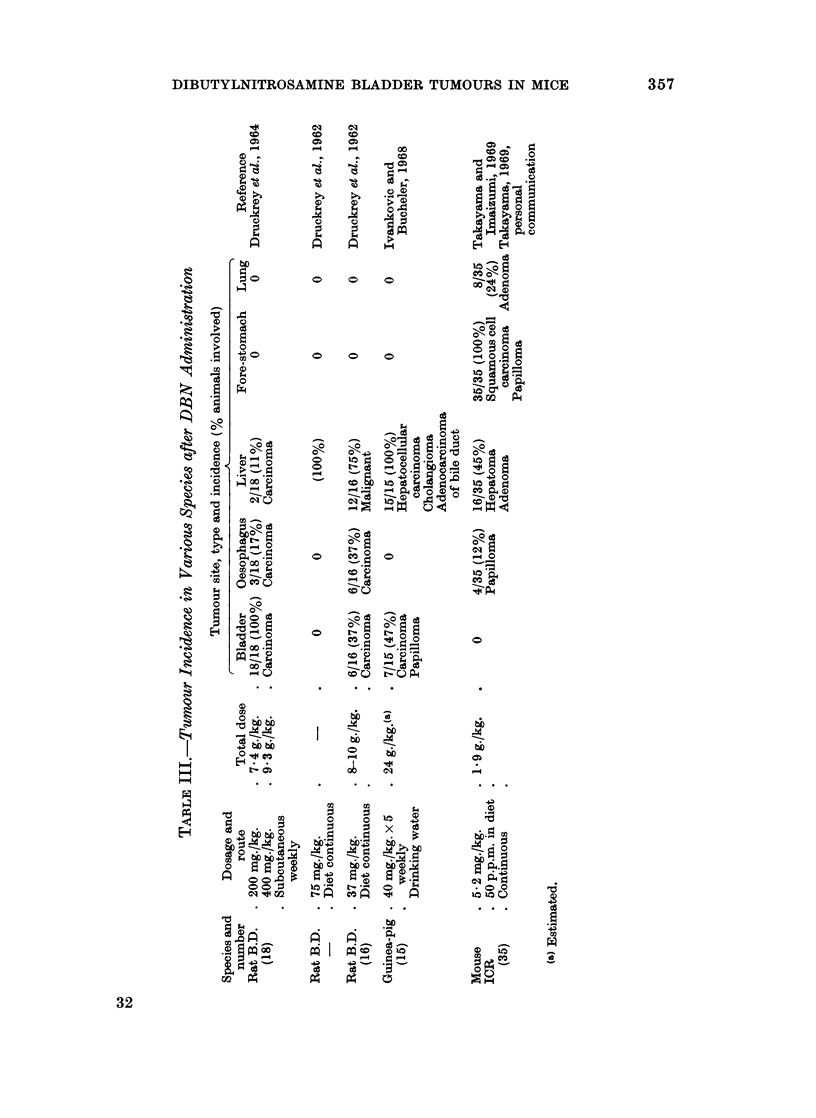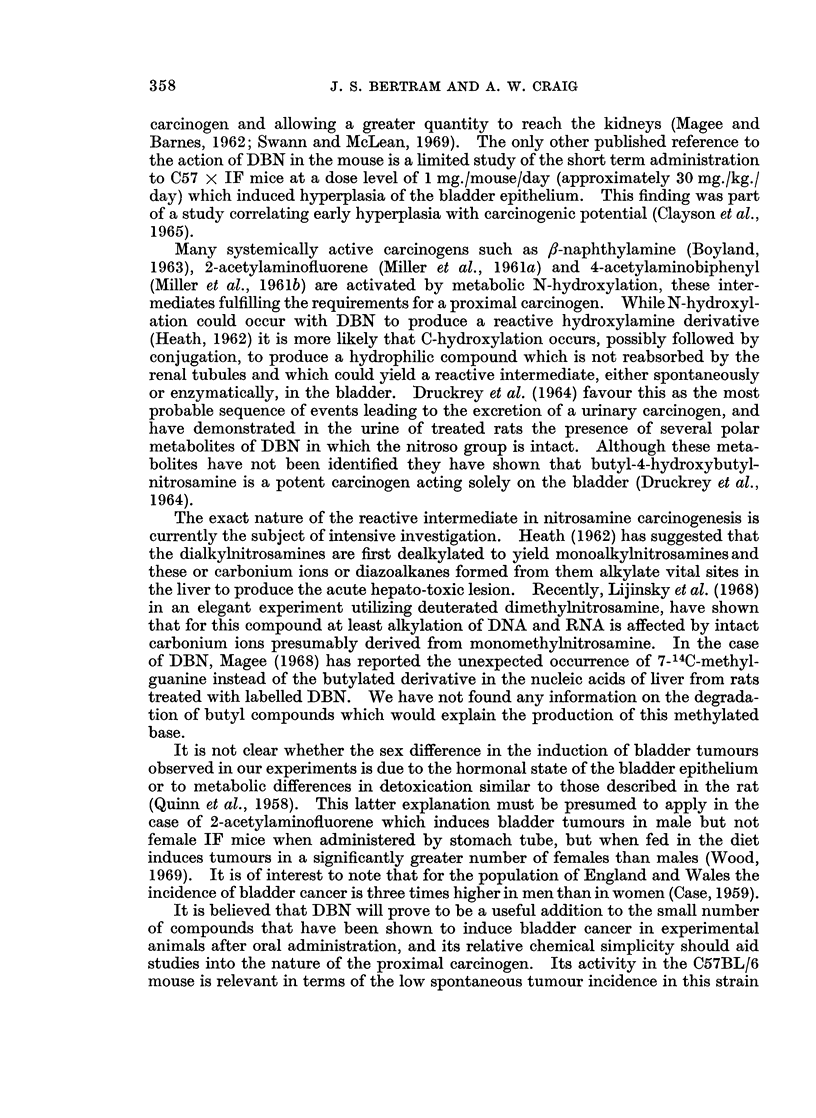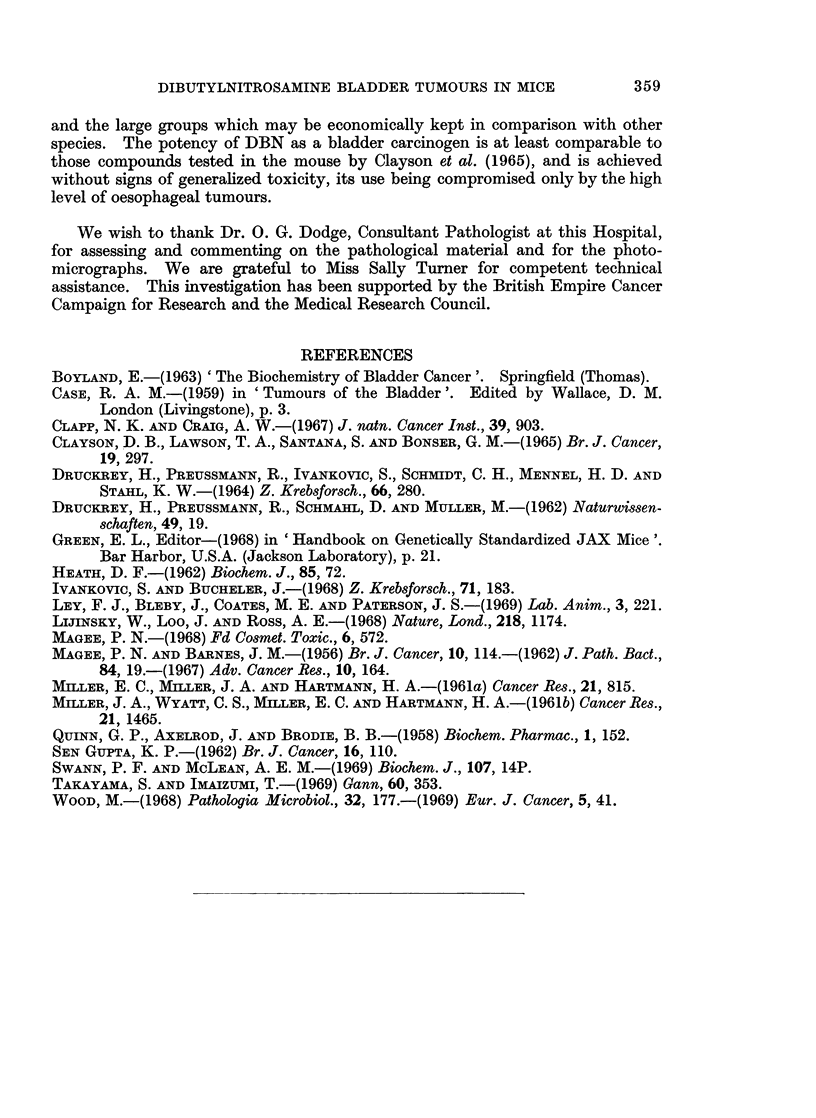# Induction of Bladder Tumours in Mice with Dibutylnitrosamine

**DOI:** 10.1038/bjc.1970.40

**Published:** 1970-06

**Authors:** J. S. Bertram, A. W. Craig

## Abstract

**Images:**


### INDUCTION OF BLADDER TUMOURS IN MICE WITH DIBUTYLNITROSAMINE

J. S. BERTRAM AND A. W. CRAIG

*From the Paterson Laboratories, Christie Hospital and Holt Radium Institute, Manchester M20 9BX*

Received for publication January 5, 1970

**SUMMARY.—Dibutylnitrosamine has been administered continuously in the drinking water to two groups of C57BL/6 mice. The first group (50 males, 50 females) received 30 mg./kg./day and the second (50 males, 50 females) 7·6 mg./kg./day. Of mice reaching autopsy squamous cell carcinoma of the bladder was found in 44/90 at the high dose and 19/89 at the low dose. Bladder tumours were found predominantly in males, the ratio of tumours in males and females being 4·4 : 1 and 8·5 : 1 at the high and low dose levels, respectively. Carcinomas and papillomas of the oesophagus were found in all but 2 of the females and all but 6 of the males. In addition 5 carcinomas of the fore-stomach in the low dose group and a total in both groups of 13 tumours of the soft palate and tongue were found. The mean cumulative doses and respective induction times for the high and low dose groups were 7·4 and 2·0 g./kg., and 240 and 260 days.**

N-NITROSO compounds were first shown to be carcinogenic by Magee and Barnes (1956) and have since been the subject of extensive research to determine both their mode of action and their structure–activity relationship. The review by Magee and Barnes (1967) gives a comprehensive coverage of this topic. The dialkyl nitrosamines predominantly produce tumours of the liver, lung, oesophagus, kidney, stomach, nasal sinus and bronchus. Dibutylnitrosamine (DBN) and its hydroxylated derivative butyl-4-hydroxybutylnitrosamine are unique among the nitrosamines in their capacity to induce bladder tumours in the rat (Druckrey *et al.*, 1962, 1964). This paper describes the carcinogenic action of DBN administered at two dose levels to C57BL/6 mice.

### MATERIALS AND METHODS

Inbred C57BL/6 mice with a low spontaneous tumour incidence (Green, 1968) were housed in groups of 10 and allowed unlimited access to food (Thomson Diet, Ley *et al.*, 1969) and drinking water containing the carcinogen. Treatment was commenced when 10–12 weeks old. The purity of the DBN used (obtained from Eastman-Kodak) was checked by thin-layer and gas liquid chromatography. Drinking water solutions were changed twice weekly and the volume drunk recorded. The total carcinogenic dose for each mouse was calculated on the assumption that individual mice within a cage consumed similar amounts of water (Clapp and Craig, 1967).

Mice were divided into two dose groups, Group A (50 males, 50 females) receiving 240 mg./litre of DBN and Group B (50 males, 50 females) 60 mg./litre of DBN. When after 197 days the majority of Group A males were grossly haematuric the carcinogen solution was replaced by drinking water for approximately

TABLE I.—*Tumour Incidence in C57BL/6 Mice after Continuous Administration of Dibutylnitrosamine in the Drinking Water*

| Group | Dose level | Sex | Number | Number with tumours | Tumour incidence (number of animals): Bladder alone | Bladder + oesophagus | Oesophagus[a] | Other |
| --- | --- | --- | --- | --- | --- | --- | --- | --- |
| A | 29·1 mg./kg./day | Male | 45 | 45 | 5 | 31 | 9 | 1 tongue papilloma[b] |
| | 30·9 mg./kg./day | Female | 45 | 45 | 0 | 8 | 37 | 1 tongue papilloma[b]<br>2 tongue papilloma } [c]<br>1 soft palate papilloma } [c] |
| B | 7·6 mg./kg./day | Male | 47 | 47 | 1 | 16 | 29 | 1 fore-stomach carcinoma }<br>1 fore-stomach carcinoma }<br>1 soft palate papilloma } [c]<br>1 tongue papilloma }<br>1 tongue carcinoma } |
| | 8·2 mg./kg./day | Female | 42 | 42 | 0 | 2 | 38 | 2 fore-stomach carcinoma }<br>1 fore-stomach carcinoma }<br>1 soft palate papilloma } [c]<br>3 tongue papilloma }<br>1 tongue carcinoma } |

[a] All cases except when in conjunction with bladder.

[b] In conjunction with bladder and oesophagus.

[c] In conjunction with oesophagus.

half the males and females of this group. In the remainder of this group and all of Group B treatment was continued until animals became moribund or died. Whenever possible a complete autopsy was performed. Tissues were fixed with 10% formalin and stained with haematoxylin and eosin for histological examination. Bladders were distended with 10% formalin by urethral cannulation before removal (Sen Gupta, 1962). After fixation the base and apex were severed to allow examination of the mucosa for tumours.

### RESULTS

Tumours were found in all mice reaching autopsy. A total of 10 animals in Group A (5 males, 5 females) and 11 animals in Group B (3 males, 8 females) were extensively cannibalized when found. Their deaths occurred at a time when lethal tumours were occurring and have been excluded from the data.

*Group A*. The mean dosage was 29·1 mg./kg./day and 30·9 mg./kg./day for the males and females respectively; details of tumour incidence are given in Table I. Bladder tumours developed in 44 animals (48%) the distribution showing a marked sex variation with 80% of the males and only 18% of the females developing tumours of this organ, a ratio of 4·4 : 1. The bladders of all 9 males with oesophageal tumours as sole site showed varying degrees of hyperplasia while many of the bladders of equivalent females were apparently normal. Oesophageal tumours developed in all but 5 of the mice. All 5 tongue and soft palate tumours were found in conjunction with oesophageal tumours and in 2 cases, were also associated with bladder tumours.

The mean tumour induction time (all sites) for the males (236 $\pm$ 25·4 days) did not differ significantly from that of the females (243 $\pm$ 24·2 days), however, bladder tumours developed in females significantly later ($P > 0{\cdot}01$) than in males (Table II). It may be speculated that had not death from oesophageal tumours supervened both sexes would have had a uniformly high bladder tumour incidence.

TABLE II.—*Tumour Induction Time for Group A and B Classified for Sex and Tumour Site*

| | | | Tumour site and induction time (days) | | |
|---|---|---|---|---|---|
| Group[a] | Sex | Number | All sites | Bladder | Oesophagus[b] |
| $A_1$ | Male | 20 | 236±20[c] | 234±22 | 241±10 |
| $A_2$ | Male | 25 | 237±29 | 234±31 | 251±7 |
| $A_1$ | Female | 21 | 243±22 | 253±13 | 240±23 |
| $A_2$ | Female | 24 | 245±26 | 253±17 | 243±28 |
| B | Male | 47 | 261±31·8 | 268±29 | 259±33 |
| | Female | 42 | 256±31·3 | 232[d] | 256±30 |

(a) $A_1$ treatment ceased after 197 days, $A_2$ treatment continued until death.
(b) All cases except when in conjunction with bladder.
(c) Mean $\pm$ standard deviation.
(d) Two mice only.

The tumour incidence and latency in those animals in which treatment was ceased after 197 days did not differ significantly from those in which treatment was continued until death (Table II). The mean total carcinogenic dose for this latter group was 6310 $\pm$ 581 mg./kg. and 7115 $\pm$ 710 mg./kg. in the males and females respectively. For all other purposes Group A has been dealt with as a whole.

*Group B*. The mean dose consumed was 7·6 mg./kg./day and 8·2 mg./kg./day for the males and females leading to a respective total carcinogenic dose of 1986 ± 199 mg./kg. and 2096 ± 236 mg./kg. Details of tumour induction are given in Table I. Bladder tumours developed in only 19 animals of this group, a sex ratio of 8·5 : 1 in favour of the males being even more pronounced than in Group A. Except for 3 mice that died early with tumours of the fore-stomach, all animals developed oesophageal tumours. In a total of 5 mice carcinoma of the fore-stomach was recorded; a tumour site not observed in Group A. No significant differences in induction times could be observed between the various tumour sites or between male and female mice (Table II).

### *Pathology*

Tumours were detected in all animals reaching autopsy. Oesophageal tumours were always multifocal and bladder tumours generally multifocal in origin. Several organs were often involved in the same animal. As far as can be judged no deaths occurred as a result of non-specific or hepato-cellular toxicity. Death most frequently resulted from respiratory obstruction of the glottal region caused by malignant or hyperplastic growth of the squamous epithelium in this region. Even large tumours of the oesophagus were rarely found to cause death, the glottis with its cartilaginous structure presumably is less able to accommodate obstructive masses. In males with the bladder as sole tumour site hydronephrosis clearly indicated renal failure as the cause of death. Similarly in those mice developing tumours of the fore-stomach, oesophageal reflux and absence of food in the alimentary tract indicated death from starvation.

Haematuria was first observed in a male from Group A after 132 days treatment and after 233 days 38 mice in Group A had shown this symptom. As mice were not consistently haematuric it seems probable that all bladder tumours were preceded by haematuria, the time interval generally elapsing between onset of symptoms and death being approximately 40 days.

Bladder tumours were frequently multifocal in origin. Squamous cell carcinoma were found in all affected bladders sometimes in association with papillomas. None of the carcinomas had penetrated further than the muscle wall of the bladder but many were infiltrating under adjacent epithelium and into the bladder lumen (Fig. 1). No prediliction for a particular site within the bladder could be determined as has been shown for 2-acetylaminofluorene in the rabbit (Wood, 1968). In cases where the carcinoma had not yet spread to involve the entire epithelium, microscopic examination revealed the epithelium to be in parts essentially normal (2–3 cells deep), progressing to hyperplastic (7–10 cells deep) often with oedema of the underlying connective tissue. Sometimes associated with this hyperplastic epithelium were found pedunculated papillomas (Fig. 2). Subcutaneous implants into normal male mice of portions of bladder carcinoma grew slowly to produce large tumours with necrotic centres.

Tumours of the oesophagus were invariably of multifocal origin and for reasons of simplicity the pharynx has been included in this classification. On autopsy the tumours were readily observed giving the oesophagus the appearance of a string of beads. These nodules were generally found to be due to large papillomas having a cauliflower-like morphology and a slender base. It was not uncommon to find up to 12 papillomas of various sizes within a single oesophagus.

Papillomas occurring in the oral cavity had a similar morphology. Squamous cell carcinomas, with the exception of 3 cases, were 1 mm. or less in diameter and it is reasonable to assume that they did not contribute to the death of the animals (Fig. 3). The hyperplasia of the squamous epithelium of the pharynx which led to the death of the majority of the experimental animals can clearly be seen in Fig. 4 which also demonstrates the abnormal state of the oesophageal epithelium contrasting strongly with the apparently normal trachea. The distribution of tumours down the length of the oesophagus appeared to be random. Although oesophageal tumours were found to occur in close proximity to the gastro-oesophageal junction, in only 5 cases were tumours found in the stomach. These were squamous cell carcinoma of the fore-stomach and were all large and apparently highly malignant. Metastases to the diaphragm and intestinal mesentery were found in 2 cases. Metastases of other tumours were not observed. Mice with bladder carcinomas generally had enlarged abdominal lymph nodes but tumour cells were not detected when they were examined histologically.

In spite of the centrolobular necrosis of the liver produced after toxic doses of DBN in rats (Heath, 1962) and mice (these laboratories, unpublished results) no pathological changes could be detected in the livers of mice treated with the dose levels reported here. In addition, despite the intimate contact of urine with the entire urinary tract, neither kidneys nor ureters were affected (neglecting hydronephrosis consequent on bladder tumour development).

### DISCUSSION

Variations in species, dose and mode of administration led to very significant changes in the carcinogenic action of DBN (Table III). Liver tumours have been reported in the rat (Druckrey *et al.*, 1962, 1964), guinea pig (Ivankovic and Bucheler, 1968) and ICR mouse (Takayama and Imaizumi, 1969) but were not observed in the present experiments. Conversely bladder tumours were not found in the male ICR mouse but occur with high frequency in the male C57BL/6 mouse; a strain with a very low spontaneous tumour incidence. The difference between these two mouse strains may simply be related to the dose levels used. In our experiments using the C57BL/6 mouse the male bladder tumour incidence falls from 80% in animals given 30 mg./kg./day to 36% at 7·5 mg./kg./day, as the dosage to the ICR mouse was only 5·24 mg./kg./day (Takayama, 1969, personal communication), this may have been below the threshold for bladder tumour induction. The mechanism by which the bladder is spared could be analogous to the situation in rats treated with dimethylnitrosamine which, although predominantly a liver carcinogen, induces kidney tumours when given in acute near toxic doses, probably by overriding the ability of the liver to metabolize the

---

### EXPLANATION OF PLATES

FIG. 1.—Edge of squamous cell carcinoma of bladder showing invasion of muscle and adjacent epithelium.

FIG. 2.—Bladder papilloma showing no invasive tendencies.

FIG. 3.—Squamous cell carcinoma of the oesophagus. The tumour (T) projects into and has practically blocked the lumen. Areas of invasion in the smooth muscle layer are arrowed.

FIG. 4.—Section through the glottal region (G) showing the occluded airway (arrowed). The normal state of the tracheal epithelium (Tr) contrasts strongly with that of the oesophagus (O) which shows hyperplasia and probable early neoplastic change.

1

2

Bertram and Craig.

3

4

TABLE III.—*Tumour Incidence in Various Species after DBN Administration*

| Species and number | Dosage and route | Total dose | Tumour site, type and incidence (% animals involved) | | | | | Reference |
|---|---|---|---|---|---|---|---|---|
| | | | Bladder | Oesophagus | Liver | Fore-stomach | Lung | |
| Rat B.D. (18) | 200 mg./kg.<br>400 mg./kg.<br>Subcutaneous weekly | 7·4 g./kg.<br>9·3 g./kg. | 18/18 (100%)<br>Carcinoma | 3/18 (17%)<br>Carcinoma | 2/18 (11%)<br>Carcinoma | 0 | 0 | Druckrey *et al.*, 1964 |
| Rat B.D.<br>— | 75 mg./kg.<br>Diet continuous | — | 0 | 0 | (100%) | 0 | 0 | Druckrey *et al.*, 1962 |
| Rat B.D. (16) | 37 mg./kg.<br>Diet continuous | 8–10 g./kg. | 6/16 (37%)<br>Carcinoma | 6/16 (37%)<br>Carcinoma | 12/16 (75%)<br>Malignant | 0 | 0 | Druckrey *et al.*, 1962 |
| Guinea-pig (15) | 40 mg./kg. × 5 weekly<br>Drinking water | 24 g./kg.[a] | 7/15 (47%)<br>Carcinoma<br>Papilloma | 0 | 15/15 (100%)<br>Hepatocellular carcinoma<br>Cholangioma<br>Adenocarcinoma of bile duct | 0 | 0 | Ivankovic and Bucheler, 1968 |
| Mouse ICR (35) | 5·2 mg./kg.<br>50 p.p.m. in diet<br>Continuous | 1·9 g./kg. | 0 | 4/35 (12%)<br>Papilloma | 16/35 (45%)<br>Hepatoma<br>Adenoma | 35/35 (100%)<br>Squamous cell carcinoma<br>Papilloma | 8/35 (24%)<br>Adenoma | Takayama and Imaizumi, 1969<br>Takayama, 1969, personal communication |

[a] Estimated.

carcinogen and allowing a greater quantity to reach the kidneys (Magee and Barnes, 1962; Swann and McLean, 1969). The only other published reference to the action of DBN in the mouse is a limited study of the short term administration to C57 $\times$ IF mice at a dose level of 1 mg./mouse/day (approximately 30 mg./kg./day) which induced hyperplasia of the bladder epithelium. This finding was part of a study correlating early hyperplasia with carcinogenic potential (Clayson *et al.*, 1965).

Many systemically active carcinogens such as $\beta$-naphthylamine (Boyland, 1963), 2-acetylaminofluorene (Miller *et al.*, 1961*a*) and 4-acetylaminobiphenyl (Miller *et al.*, 1961*b*) are activated by metabolic N-hydroxylation, these intermediates fulfilling the requirements for a proximal carcinogen. While N-hydroxylation could occur with DBN to produce a reactive hydroxylamine derivative (Heath, 1962) it is more likely that C-hydroxylation occurs, possibly followed by conjugation, to produce a hydrophilic compound which is not reabsorbed by the renal tubules and which could yield a reactive intermediate, either spontaneously or enzymatically, in the bladder. Druckrey *et al.* (1964) favour this as the most probable sequence of events leading to the excretion of a urinary carcinogen, and have demonstrated in the urine of treated rats the presence of several polar metabolites of DBN in which the nitroso group is intact. Although these metabolites have not been identified they have shown that butyl-4-hydroxybutylnitrosamine is a potent carcinogen acting solely on the bladder (Druckrey *et al.*, 1964).

The exact nature of the reactive intermediate in nitrosamine carcinogenesis is currently the subject of intensive investigation. Heath (1962) has suggested that the dialkylnitrosamines are first dealkylated to yield monoalkylnitrosamines and these or carbonium ions or diazoalkanes formed from them alkylate vital sites in the liver to produce the acute hepato-toxic lesion. Recently, Lijinsky *et al.* (1968) in an elegant experiment utilizing deuterated dimethylnitrosamine, have shown that for this compound at least alkylation of DNA and RNA is affected by intact carbonium ions presumably derived from monomethylnitrosamine. In the case of DBN, Magee (1968) has reported the unexpected occurrence of 7-$^{14}$C-methylguanine instead of the butylated derivative in the nucleic acids of liver from rats treated with labelled DBN. We have not found any information on the degradation of butyl compounds which would explain the production of this methylated base.

It is not clear whether the sex difference in the induction of bladder tumours observed in our experiments is due to the hormonal state of the bladder epithelium or to metabolic differences in detoxication similar to those described in the rat (Quinn *et al.*, 1958). This latter explanation must be presumed to apply in the case of 2-acetylaminofluorene which induces bladder tumours in male but not female IF mice when administered by stomach tube, but when fed in the diet induces tumours in a significantly greater number of females than males (Wood, 1969). It is of interest to note that for the population of England and Wales the incidence of bladder cancer is three times higher in men than in women (Case, 1959).

It is believed that DBN will prove to be a useful addition to the small number of compounds that have been shown to induce bladder cancer in experimental animals after oral administration, and its relative chemical simplicity should aid studies into the nature of the proximal carcinogen. Its activity in the C57BL/6 mouse is relevant in terms of the low spontaneous tumour incidence in this strain and the large groups which may be economically kept in comparison with other species. The potency of DBN as a bladder carcinogen is at least comparable to those compounds tested in the mouse by Clayson *et al.* (1965), and is achieved without signs of generalized toxicity, its use being compromised only by the high level of oesophageal tumours.

We wish to thank Dr. O. G. Dodge, Consultant Pathologist at this Hospital, for assessing and commenting on the pathological material and for the photomicrographs. We are grateful to Miss Sally Turner for competent technical assistance. This investigation has been supported by the British Empire Cancer Campaign for Research and the Medical Research Council.

### REFERENCES

BOYLAND, E.—(1963) ' The Biochemistry of Bladder Cancer '. Springfield (Thomas).

CASE, R. A. M.—(1959) in ' Tumours of the Bladder '. Edited by Wallace, D. M. London (Livingstone), p. 3.

CLAPP, N. K. AND CRAIG, A. W.—(1967) *J. natn. Cancer Inst.*, **39**, 903.

CLAYSON, D. B., LAWSON, T. A., SANTANA, S. AND BONSER, G. M.—(1965) *Br. J. Cancer*, **19**, 297.

DRUCKREY, H., PREUSSMANN, R., IVANKOVIC, S., SCHMIDT, C. H., MENNEL, H. D. AND STAHL, K. W.—(1964) *Z. Krebsforsch.*, **66**, 280.

DRUCKREY, H., PREUSSMANN, R., SCHMAHL, D. AND MULLER, M.—(1962) *Naturwissenschaften*, **49**, 19.

GREEN, E. L., Editor—(1968) in ' Handbook on Genetically Standardized JAX Mice '. Bar Harbor, U.S.A. (Jackson Laboratory), p. 21.

HEATH, D. F.—(1962) *Biochem. J.*, **85**, 72.

IVANKOVIC, S. AND BUCHELER, J.—(1968) *Z. Krebsforsch.*, **71**, 183.

LEY, F. J., BLEBY, J., COATES, M. E. AND PATERSON, J. S.—(1969) *Lab. Anim.*, **3**, 221.

LIJINSKY, W., LOO, J. AND ROSS, A. E.—(1968) *Nature, Lond.*, **218**, 1174.

MAGEE, P. N.—(1968) *Fd Cosmet. Toxic.*, **6**, 572.

MAGEE, P. N. AND BARNES, J. M.—(1956) *Br. J. Cancer*, **10**, 114.—(1962) *J. Path. Bact.*, **84**, 19.—(1967) *Adv. Cancer Res.*, **10**, 164.

MILLER, E. C., MILLER, J. A. AND HARTMANN, H. A.—(1961*a*) *Cancer Res.*, **21**, 815.

MILLER, J. A., WYATT, C. S., MILLER, E. C. AND HARTMANN, H. A.—(1961*b*) *Cancer Res.*, **21**, 1465.

QUINN, G. P., AXELROD, J. AND BRODIE, B. B.—(1958) *Biochem. Pharmac.*, **1**, 152.

SEN GUPTA, K. P.—(1962) *Br. J. Cancer*, **16**, 110.

SWANN, P. F. AND MCLEAN, A. E. M.—(1969) *Biochem. J.*, **107**, 14P.

TAKAYAMA, S. AND IMAIZUMI, T.—(1969) *Gann*, **60**, 353.

WOOD, M.—(1968) *Pathologia Microbiol.*, **32**, 177.—(1969) *Eur. J. Cancer*, **5**, 41.